# Comparison of IgG against plastic resin in workers with and without chemical dermatitis

**DOI:** 10.1186/s12889-015-2302-4

**Published:** 2015-09-21

**Authors:** Toshihiro Kawamoto, Mayumi Tsuji, Toyohi Isse

**Affiliations:** Department of Environmental Health, University of Occupational and Environmental Health, 1-1 Iseigaoka, Yahatanishi-ku, Kitakyushu 807-8555 Japan

**Keywords:** Allergy, IgG, Plastic resin, Dermatitis, Dot blotting, Worker

## Abstract

**Background:**

There are many chemical sensitizers which cause allergy in the surrounding environment. However, the identification of substances causing allergy is difficult. We developed a new method to detect IgG which reacts against many kinds of chemical-human serum albumin (HSA) adducts at the same time. In this study, the diagnostic significance of the IgG was studied among workers of a company where a mass outbreak of chemical dermatitis had occurred after changing a plastic resin to a new one.

**Methods:**

Eleven workers who handled the new plastic resin and suffered from dermatitis (case) and 9 workers who also handled the same resin in the same company but were free from dermatitis (control) were the subjects. Immunological dot blotting was carried out to detect serum IgG using originally prepared diagnostic antigens, comprising a mixture of HSA and the plastic resin or its components under various conditions.

**Results:**

IgG against the plastic resin in use was detected in all workers who suffered from dermatitis. The prevalence of the IgG against the plastic resin was significantly higher in workers with than in those without dermatitis. On the other hand, IgG against its components (bisphenol A diglycidyl ether, *m*-xylylenediamine and butyl 2,3-epoxypropyl ether) was detected in a few workers with dermatitis.

**Discussion:**

This suggests that IgG against chemical-HSA adduct reflects not only exposure but also causative chemicals of dermatitis. Our method to use a material itself as a hapten is practical and useful in the occupational field.

**Conclusion:**

It is suggested that IgG against chemicals is a useful marker of chemicals inducing dermatitis.

**Electronic supplementary material:**

The online version of this article (doi:10.1186/s12889-015-2302-4) contains supplementary material, which is available to authorized users.

## Background

Occupational contact dermatitis caused by epoxy resin is an important health concern due to the wide diffusion of products containing this powerful hapten [1, 2]. The prevalence of epoxy resin sensitization was 0.89 % in 19,088 consecutive patients with symptoms and/or signs of suspected allergic dermatitis in Northeastern Italy form 1996 to 2010 [1]. Positive patch test reaction to epoxy resin was found in 1.3 % of 20,808 consecutive dermatitis patients in Denmark [3]. The American Conference of Governmental Industrial Hygienists (ACGIH) gave the designation “SEN” to 1,2-epoxypropane; propylene oxide (CAS#: 75-56-9) and 2,3-epoxypropyl phenyl ether (CAS#: 122-60-1), referring “the potential for an agent to produce sensitization, as confirmed by human or animal data”. 1-Allyloxy-2,3-epoxypropane; allyl glycidyl ether (CAS#: 106-92-3), polymer of 4,4-isopropylidenediphenol & 1-chloro-2,3-epoxypropane (liquid); diglycidylether of BPA; bisphenol A type epoxy resin (liquid) (CAS#: 25068-38-6), 1,3,5-tris(2,3-epoxypropyl)-1,3,5-triazine-2,4,6-(1H,3H,5H)-trione; 3,5-triglycidyl-S-triazinetrione (CAS#:2451-62-9) and 2,3-epoxypropyl methacrylate; glycidyl methacrylate; GMA (CAS#:106-91-2) are classified as “R43: May cause sensitization by skin contact” by the European Unions [4, 5]. Other than epoxy resins, some materials of urethane resins, acrylic resins and so on are also designated as sensitizers.

Even though it is known that plastic resin materials cause sensitization, the identification of individual chemicals causing dermatitis is very difficult because there are so many sensitizing chemical substances other than plastic resin materials surrounding us in our everyday lives. Patch test and skin prick tests are commonly used to diagnose causative chemicals for allergic dermatitis. However, these tests have a risk to sensitize patients [6].

We developed a new method to detect serum IgG or IgE against many kinds of chemical-HSA adducts at the same time. In this study, we measured IgG and IgE against a plastic resin and its component using this method among workers of a house foundation repair and reinforcement company where a mass outbreak of allergic dermatitis had taken place just after they had started to use a new plastic (epoxy) resin.

## Methods

### Case

A house foundation repair and reinforcement company changed a plastic (epoxy) resin to a new one in 2007. Most workers engaged in repair and reinforcement of house basements started to complain of pruritus, redness, and swelling on the face, neck, and upper limbs. The onset was just 1 or 2 hours after the initial use of the new plastic resin in some workers and 2 or 3 weeks later in others. On the other hand, some workers who were engaged in the same work showed no skin problems. In this study, we enrolled 11 workers who had consulted dermatologists and been diagnosed with allergic contact dermatitis. They were recognized as an occupational disease by Labor Standards Inspection Office. Nine workers, who had engaged in the same work in the same company, but had not suffered from dermatitis about six months after the onset, were also enrolled as controls. This study was approved by the Institutional Ethics Committee at the University of Occupational and Environmental Health (#05-57). Written consent was obtained from all participants.

### Chemicals

The plastic resin which is suspected to have caused the mass outbreak of dermatitis was kindly provided by the house foundation repair and reinforcement company. The plastic resin is called Product-X in this paper. Bisphenol A diglycydil ether (BADGE), *m*-xylylenediamine (XDA) and butyl 2,3-epoxypropyl ether (BEE) were purchased from Wako Chemical Co. Human serum albumin (HSA: > = 99 %, essentially globulin-free) was obtained from the Sigma Corporation. Human IgG (chromatographically purified) and horseradish peroxidase (HRP) conjugated goat anti-human IgG (AFFINITY PURIFIED SECONDARY ANTIBODY) were purchased from ZYMED® Laboratories and Millipore, respectively. Human IgE myeloma, and horseradish peroxidase (HRP) labeled goat anti-Human IgE (epsilon) antibody were obtained from Calbiochem® and Kirkegaard & Perry Laboratories, Inc. (KPL). Nitrocellulose (NC) blotting membrane was Amersham Hybond ECL (Cat No. RPN2020D) made in Germany.

### Components of Product-X

Product-X is an epoxy resin composed of two agents, a base resin and a hardener. According to a safety data sheet (SDS), the base resin contains bisphenol A type epoxy resin (50 – 60 %), silica (1 – 5 %), and titanium　oxide (1 – 5 %). The　hardener　contains　*m*-xylylenediamine (<8 %) and phenol (1-5 %). Besides these, butyl 2,3-epoxypropyl　ether,　carbon　black,　aramid　fiber, and　inoraganic　fibers are contained in the base resin, and　isophorondiamine, benzylalcohol, and ethyltrismethane in the hardener based on an inquiry by the house foundation repair and reinforcement company to the product manufacturer.

### Preparation of diagnostic antigens, chemical-HSA adducts

There kinds of pH buffer were prepared. Two of them were 100 mM sodium phosphate buffer at pH 7.4 and 8.0. The other was 100 mM sodium carbonate buffer at pH 9.2. BADGE, XDA, and BEE were mixed with 100 μM of human serum albumin (HSA) in pH 7.4, 8.0, and 9.4 buffer solutions. BADGE, XDA and BEE were mixed with 100 μM HSA in the three different pH buffer solutions at the ratios (Chemical : HSA) were 1:1, 12.5:1, 25:1, 50:1, 100:1, 200:1, and 1,000:1 in molecular bases. The base resin of Product-X was also mixed with HSA in the buffer solutions with final concentrations of 0.01, 0.1, 0.25, 0.5, 1, 2, and 4 % (w/v). The mixing was carried out at 25 °C for 3 hours and the supernatant was dispensed in a small volume and stored at −80 °C until analyses. The chemical-HSA adducts served as diagnostic antigens.

### Dot blotting

One μL of the diagnostic antigens was blotted on a NC membrane. After blocking with blocking buffer (Nacalai Tesque, sp05150-45), the membrane was treated with patients’ serum which was diluted 200 times with phosphate-buffered saline containing 0.1 % tween 20 (PBST) for 60 min. After washing with PBST twice, the membrane was treated with horseradish peroxidase (HRP)-conjugated goat anti human-IgG or horseradish peroxidase (HRP)-labeled goat anti-Human IgE (epsilon). The chemiluminescence was measured based on a light capture (ATTO: AE-6972/C/FC) with Amersham ECL Western blotting detection reagents (GE Healthcare, RPN2106). The result was determined positive with chemiluminescence of at least one dot spot.

### Measurements of immunological parameters

Total IgE, total IgG, cortisol, IFN-gamma, IL-1beta, IL-2, IL-5, IL-4, IL-6, and the CD4/CD8 ratio in serum were measured by SRL, Inc., Tokyo, Japan (Additional file 1). Specific serum IgE antibody was measured with the multiple allergen simultaneous test (MAST)-26 chemiluminescent assay systems (Hitachi Chemical Co., Tokyo, Japan) [7].

## Results

### Study participants

Subject characteristics regarding the age, smoking status, drinking habit, and allergic history are summarized in Table 1. No significant differences were observed between the two worker groups with and without dermatitis.

**Table 1:** Profiles of the study subjects

	Dermatitis	
	Yes	No
Number of subjects	11	9
Age	43.5 ± 3.3	41.6 ± 3.9
Number of current smokers (%)	8 (72.3 %)	7 (77.8)
Number of habitual drinkers (%)	7 (63.6)	6 (66.7)
Allergic history (%)	2 (18.2)	4 (44.4)

### Specific IgE against inhalant and food antigens and CD4/CD8 ratio

The IgE levels against common allergens were compared between the two worker groups with and without dermatitis. No significant differences were observed between the two groups except for *dermatophagoides farinae* and shrimp. However, the median and range of IgE levels against *dermatophagoides farinae* and shrimp were higher in the workers without than in those with dermatitis. Serum cortisol and cytokines (IL-1beta, IL-2, IL-5, IL- 4, and IL-6) did not show any differences between the two groups. CD4/CD8 ratio in workers with dermatitis was not different from that in those without dermatitis. These results indicate that there is no association between the onset of dermatitis and atopy (Table 2).

**Table 2:** Specific IgE against inhalant and food antigens and CD8/CD4

		Dermatitis				
		Yes		No		
	Units	Median	(Range)	Median	(Range)	
Nonspecific IgE	IU/mL	84.3	(15.1 - 442)	76.6	(6.9 – 1,930)	n.s.
*Dermatophagoides farinae*	lumicount	0.44	(0–68.3)	20.8	(0.24 - 99.9)	*p* < 0.05
House dust	lumicount	0.32	(0–3.43)	0.38	(0–19.1)	n.s.
Cat	lumicount	0.16	(0–5.19)	0.23	(0–1.46)	n.s.
Dog	lumicount	0.28	(0.07 - 0.56)	0.43	(0–24.1)	n.s.
Timothy grass	lumicount	0.32	(0.16 - 18.4)	0.8	(0–99.6)	n.s.
Vernal grass	lumicount	0.09	(0–12.7)	0.33	(0–75.3)	n.s.
Ragweed mix	lumicount	0.06	(0–0.16)	0.02	(0–4.46)	n.s.
Mugwort	lumicount	0.08	(0–1.93)	0	(0–96.4)	n.s.
Japanese cedar	lumicount	0.72	(0–81.5)	0.65	(0–49.8)	n.s.
Penicillin	lumicount	0	(0–0.07)	0	(0–2.38)	n.s.
Cladosporium	lumicount	0	(0–0.25)	0.01	(0–2.08)	n.s.
Candida	lumicount	0	(0–0.33)	0	(0–1.17)	n.s.
Alternaria	lumicount	0	(0–0.12)	0	(0–0.77)	n.s.
Aspergillus	lumicount	0.13	(0–0.32)	0.3	(0–3.99)	n.s.
Wheat	lumicount	0	(0–0.1)	0	(0–1.56)	n.s.
Soy bean	lumicount	0.05	(0–0.21)	0.02	(0–2.58)	n.s.
Rice	lumicount	0.02	(0–0.14)	0	(0–2.93)	n.s.
Tuna	lumicount	0.02	(0–0.26)	0.11	(0–1.26)	n.s.
Salmon	lumicount	0.05	(0–0.23)	0.09	(0–0.59)	n.s.
Shrimp	lumicount	0	(0–0.27)	0.18	(0–0.98)	*p* < 0.05
Crab	lumicount	0.04	(0–0.22)	0.13	(0–9.48)	n.s.
Cheddar cheese	lumicount	0.01	(0–0.27)	0	(0–0.21)	n.s.
Milk	lumicount	0.08	(0–0.42)	0	(0–1.13)	n.s.
Beef	lumicount	0	(0–0.16)	0	(0–0.8)	n.s.
Chicken	lumicount	0	(0–0.08)	0.01	(0–1.02)	n.s.
Egg white	lumicount	0.12	(0–0.36)	0.08	(0–0.53)	n.s.
Cortisol	ug/dL	4.8	(2.5 - 12)	5	(3.5 - 19.4)	n.s.
IFN-gamma	IU/mL	Under detection limit		Under detection limit		
IL-1beta	pg/mL	5	(5–5)	5	(5–13)	n.s.
IL-2	U/mL	Under detection limit		Under detection limit		
IL-5	pg/mL	6.2	(2.5 - 9)	5.3	(2.5 - 9)	n.s.
IL-4	pg/mL	10.8	(3.6 - 31.1)	7.2	(3.6 - 11.1)	n.s.
IL-6	pg/mL	1.2	(0.6 - 18.6)	1.3	(8–41.2)	n.s.
CD8(+)CD4(+)	%	1.5	(0.4 - 5.5)	0.8	(0.4 - 1.9)	n.s.
CD8(+)CD4(−)	%	26.8	(14.5 - 37.7)	25.3	(15.2 - 35.1)	n.s.
CD8(−)CD4(+)	%	49.3	(31.8 - 57.8)	47.7	(43.7 - 62.2)	n.s.
CD8(−)CD4(−)	%	21.7	(15.1 - 37.2)	25.3	(16.1 - 27.5)	n.s.
CD4/CD8 ratio		1.77	(1.04 - 3.11)	1.92	(1.23 - 4.01)	n.s.

Significance was calculated using Wilcoxon rank-sum (Mann–Whitney) test

*Lumicount by multiple allergen simultaneous test (MAST)-26 chemiluminescent assay systems [7, 12]

### Detection of IgG which reacts with chemical-HSA adduct

**Fig. 1:**
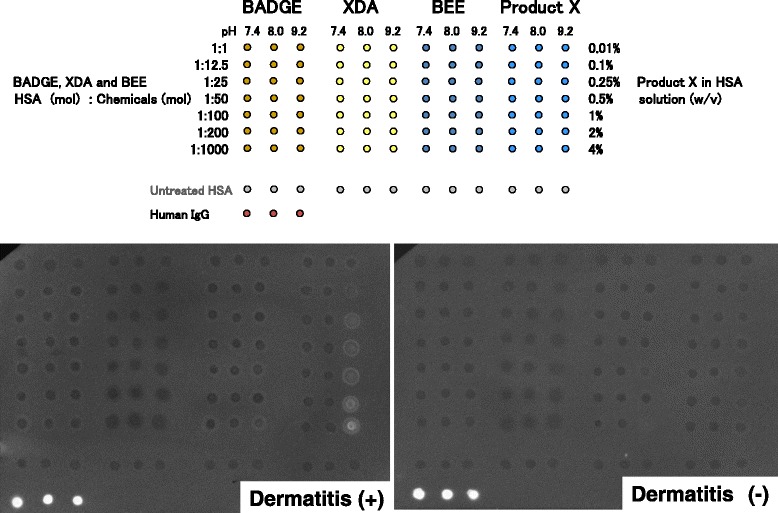
Immuno-dot blotting for detecting IgG which reacts with chemical-HSA adducts in human serum. A mixture of HSA (100 μM) and chemicals in buffer at pH 7.4, 8.0, or 9.2 was blotted on an NC membrane in the order from a low mixture rate (top) to high mixture rate (bottom), BADGE, XDA, or BEE : HSA in molecular bases ranged from 1, 12.5:1, 50:1, 100:1, 200:1, and 1,000:1. Product-X ranged from 0.01, 0.1, 1.25, 0.5, 1, 2, 4 %(w/v) in 100 μM HSA solution

Figure 1 shows the results of dot blotting. The serum from a worker with dermatitis reacted with the dot spots of Product-X high concentration at pH9.2. However, HSA-BADGE, −XDA, and -BEE antigens did not react with the serum. This indicated that the worker had IgG against Product-X-HSA adduct. On the other hand, serum from a worker without dermatitis did not react with any of BADGE, XDA, BBE, or Product-X.

### Prevalence rates of IgG and IgE which reacts with chemical-HSA adduct in the workers

The rates of workers showing IgG and IgE against BADGE, XDA, BEE, and Product-X are shown in Table 3. No IgE was detected in any workers who had handled Product-X. However, a few workers had IgG against the components of Product-X, i.e., BADGE, XDA, or BEE. All workers who suffered from dermatitis had IgG against Product-X. Although 5 (55.6 %) of the workers without dermatitis also had IgG against Product-X, there was a significant difference (p < 0.05) in the prevalence rates of the IgG between the two worker groups with and without dermatitis. The sensitivity was 100 % (11/11) and the specificity was 44.4 % (4/9).

**Table 3:** Positive rates of specific IgG and IgE against plastic resins

	Dermatitis		
	Yes (N = 11)	No (N = 9)	
IgE against BADGE	0 (0.0 %)	0 (0.0 %)	n.s.
IgE against XDA	0 (0.0)	0 (0.0)	n.s.
IgE against BEE	0 (0.0)	0 (0.0)	n.s.
IgE against Product-X	0 (0.0)	0 (0.0)	n.s.
IgG against BADGE	3 (27.3)	0 (0.0)	n.s.
IgG against XDA	1 (9.1)	0 (0.0)	n.s.
IgG against BEE	1 (9.1)	0 (0.0)	n.s.
IgG against Product-X	11 (100)	5 (55.6)	*p* < 0.05

Significance was calculated using Chi-square test

## Disccusion

The results of specific IgE levels against inhalants and food antigens, cytokine levels, and the CD4/CD8 ratio did not indicate an association of dermatitis with atopy. All workers with dermatitis had been diagnosed with allergic dermatitis by dermatological specialists in the area. However, the patients had not undergone a patch test or skin prick test in the dermatological clinics because such tests occasionally exaggerate patients’ dermatitis [6]. Although allergic dermatitis would be supposed from their history, it was impossible to diagnose as allergic dermatitis. Their dermatitis improved after ceasing to use Product-X.

The IgG which reacts with chemical-HSA adducts was detected in workers with dermatitis in this study. The diagnostic significance of the IgG on dermatitis is not clear at present. Vojdani et al. [8] reported high IgG and IgM levels against formaldehyde, terimellitic anhydride, phthalic anhydride, and a benzene ring among 289 chemical exposed workers in computer manufacturing plants. Pronk et al. [9] studied serum IgG and IgE against hexamethylene diisocyanate (HDI) in relation to exposure and respiratory symptoms in 581 workers in the spray-painting industry, and concluded that the IgE was found in only a minority of symptomatic individuals, and the IgG seems to be merely an indicator of exposure. Wisnewski et al. [10] demonstrated elevated HDI-specific serum IgG levels in aircraft painters, but it was not associated with atopy, asthma, or other demographic information. They concluded that the IgG provides a practical biomarker to aid in exposure surveillance and ongoing industrial hygiene efforts.

In this study, the prevalence of IgG against Product-X in workers with dermatitis was 100 %, being significantly higher than in those without dermatitis (p < 0.05). This suggests that IgG against chemical-HSA adduct reflects not only exposure but also causative chemicals of dermatitis. Although IgG against Product-X was positive in all workers with dermatitis, the prevalence rate of IgG against Product-X components, BADGE, XDA, and BEE, were much smaller. As mentioned in “Introduction”, the SDS describes very few components of Product-X and most components of Product-X appear to be proprietary. Possible reasons are that the workers might have IgG against such proprietary components or these components might interact with each other to produce neo-antigens. With such an SDS, it is impossible to identify causative chemicals for dermatitis. However, our method to use a material itself as a hapten is practical and useful in the occupational field. Our method makes it possible to evaluate potential exposure to plastic resins and identify causative materials.

The environment surrounding us has become quite different from what it used to be. Furniture, stationary, floors, and walls in our houses and offices are coated with artificial polymers. The Japanese government started a large-scale and long-term birth cohort study (the Japan Environment and Children’s Study: JECS) to elucidate environmental factors that affect children’s health and development [11]. IgG against chemicals measurement which was developed in this study will be useful not only to evaluate exposure to plastics resins, but also to suggest suspicious chemicals causing allergy.

## Conclusions

IgG which reacts with chemical-HSA adduct was analyzed in serum from workers who had handled a plastic resin in the same company. The prevalence of IgG against the plastic resin was significantly higher in workers who suffered from dermatitis than in workers without dermatitis. It is suggested that IgG against chemicals is a favorable marker of causative chemicals for chemically induced dermatitis.

## Additional file

Additional file 1:
**Details of the study subjects.** (PDF 125 kb)

## Abbreviations

BADGE: Bisphenol A diglycidyl ether
BEE: Butyl 2,3-epoxypropyl ether
HDI: Hexamethylene diisocyanate
HSA: Human serum albumin
NC: Nitrocellulose
SDS: Safety data sheet
XDA: Xylylenediamine
