# Supplementary material for: Comparison of IgG against plastic resin in workers with and without chemical dermatitis
Source: BMC Public Health. 2015 Sep 21;15:930. doi: 10.1186/s12889-015-2302-4 (PMC4576395; doi:10.1186/s12889-015-2302-4)
Supplement: Additional file 1: — Details of the study subjects. (PDF 125 kb) [file 12889_2015_2302_MOESM1_ESM.pdf]

ID	Age	Gender	Smoking	Alcohol	History	Allergic Dis
1	34	male	Ex	No	Allergic Rhinitis?	1
2	41	male	Yes	Habitual	—	0
3	35	male	Yse	Habitual	Atopic dermatitis	1
4	35	male	Yes	Habitual	—	0
5	55	male	Yes	Habitual	—	0
6	57	male	No	Habitual	—	0
7	56	male	Yes	Habitual	—	0
8	37	male	Yes	Habitual	—	0
9	34	male	No	No	Pollin allergy	1
10	33	male	Yes	Habitual	—	0
11	40	male	Yes	No	—	0
12	54	male	Yes	Habitual	—	0
13	32	male	Yes	No	Allergic Rhinitis	1
14	29	male	Yes	No	—	0
15	33	male	Yes	Habitual	—	0
16	35	male	Yes	Habitual	—	0
17	37	male	Yes	Habitual	Allergic to shellfish	1
18	58	male	No	No	Allergic to shellfish	1
19	60	male	Yes	No	—	0
20	58	male	No	Habitual	—	0

Dermatitis Y/N	Usage of Product X	IgG against Product X	IgG against BADGE	IgG against XDA	IgG against BEE	IgE against Product X
1	1	1	1	0	0	0
1	1	1	0	0	0	0
0	1	0	0	0	0	0
0	1	1	0	0	0	0
0	1	1	0	0	0	0
1	1	1	0	0	0	0
1	1	1	0	0	0	0
1	1	1	0	0	0	0
1	1	1	0	0	0	0
1	1	1	0	0	0	0
1	1	1	1	1	0	0
1	1	1	0	0	0	0
0	1	0	0	0	0	0
0	1	1	0	0	0	0
1	1	1	0	0	0	0
0	1	1	0	0	0	0
0	1	0	0	0	0	0
0	1	0	0	0	0	0
1	1	1	1	0	1	0
0	1	1	0	0	0	0

IgE against BADGE	IgE against XDA	IgE against BEE	Dermatophagoides farinae	House dust	Cat	Dog	Timothy grass	Vernal grass
0	0	0	68.3	3.43	0.27	0.29	0.73	0.31
0	0	0	0.76	0.11	0	0.21	18.4	12.7
0	0	0	99.8	14	1.46	24.1	2.24	1.86
0	0	0	70.5	2.88	0	0.35	0.55	0.25
0	0	0	0.69	0.38	0.58	1.7	8.2	11.1
0	0	0	0	0	0	0.07	0.22	0.1
0	0	0	0	0.09	0.16	0.47	0.23	0
0	0	0	22.1	0.45	0	0.48	0.26	0
0	0	0	1.89	0.42	5.19	0.28	0.35	0.09
0	0	0	0.44	0.37	0.24	0.12	0.16	0.04
0	0	0	0.27	0.41	1.24	0.33	0.42	0.02
0	0	0	0.31	0.32	0.22	0.27	0.35	0.12
0	0	0	20.8	0.84	0.23	0.19	0.27	0.06
0	0	0	26.4	0	0	0	0	0
0	0	0	0.31	0.06	0.08	0.17	0.16	0
0	0	0	0.67	0.07	0.27	0.55	1.24	0.33
0	0	0	2.26	0	0	0	0	0
0	0	0	99.9	19.1	0.83	1.59	99.9	75.3
0	0	0	0.44	0.12	0	0.56	0.32	0.14
0	0	0	0.24	0.12	0	0.43	0.8	0.94

Ragweed mix	Mugwort	Japanese cedar	Penicillin	Cladosporium	Candida	Alternaria	Aspergillus	Wheat
0.07	0.1	1.21	0	0.06	0.1	0	0.14	0
0	0.08	0.72	0	0	0	0	0.14	0
4.06	96.4	31.1	2.38	2.08	1.17	0	3.99	1.56
0	0	0.65	0	0	0	0	0	0
4.46	53.1	49.8	0.55	0.33	1.13	0.77	0.75	1.47
0	0	0.07	0	0.05	0	0.04	0.12	0.1
0	0	0.02	0	0	0.01	0	0.03	0.05
0.09	0.09	8.53	0	0	0	0	0.08	0
0.06	0.06	81.5	0.07	0.25	0.33	0.08	0.32	0.09
0.06	0.12	0	0.05	0	0.06	0.03	0.23	0.09
0	1.93	27.9	0	0	0	0	0	0
0.1	0.24	0.1	0.07	0.09	0.09	0.12	0.17	0
0.09	0.08	0	0	0.01	0	0.03	0.11	0
0	0	0	0	0	0	0	0	0
0	0	2.48	0	0	0	0	0.07	0
0.02	0	1.13	0.11	0	0.09	0.22	0.47	0.14
0	0	0	0	0.14	0	0	0.12	0
0.62	0.45	0.58	0.44	0.24	0.22	0.31	0.69	0.28
0.16	0	0	0	0	0	0	0.13	0
0.01	0	1.38	0	0	0	0	0.3	0

Soy bean	Rice	Tuna	Salmon	Shrimp	Crab	Cheddar cheese	Milk	Beef
0.13	0.08	0.13	0.05	0.04	0.2	0.27	0.42	0.14
0	0.14	0	0	0	0	0	0.12	0
1.37	1.11	1.26	0.59	0.98	6.52	0.21	1.13	0.8
0	0	0.02	0.09	0.01	0.1	0	0	0
2.58	2.93	0.62	0.55	0.58	0.37	0.14	0.35	0.76
0.05	0.03	0	0.09	0.04	0	0.15	0	0.16
0	0.02	0	0.05	0	0.07	0.01	0.05	0
0	0	0.09	0	0	0	0	0	0
0.07	0.08	0.02	0.22	0.17	0.14	0.15	0.15	0
0.21	0	0.19	0.07	0.27	0.04	0.06	0.27	0.09
0	0	0	0.03	0	0	0	0	0
0.13	0.05	0.06	0.23	0.05	0.22	0.01	0.08	0.08
0.02	0	0.11	0.05	0.07	0.13	0	0	0
0	0	0	0	0	0	0	0	0
0	0	0	0	0	0	0	0	0
0.11	0.06	0.16	0.01	0.18	0.12	0.1	0.14	0.01
0	0	0	0	0.91	9.48	0	0	0
0.29	0.38	0.52	0.47	0.28	2.01	0.05	0.21	0.12
0.05	0	0.26	0.02	0	0.18	0	0.09	0
0	0	0.02	0.15	0	0	0	0	0

Chicken	Egg white	Cortisol	IgE Non-specific	IFNgamma (human)	IL-1beta	IL-2	IL-5	IL-4
0.07	0.19	2.6	215	<DL	5	<DL	6.2	31.1
0	0	4.2	424	<DL	5	<DL	5.3	19.8
1.02	0.53	4.1	1930	<DL	13	<DL	2.5	5.5
0.01	0.08	5	76.6	<DL	5	<DL	2.5	8
0.89	0.34	5.8	1030	<DL	5	<DL	5.2	9.8
0	0.12	4.8	132	<DL	5	<DL	6.2	21.9
0.03	0.12	2.5	15.1	<DL	5	<DL	8	11.6
0	0.05	2.8	69.9	<DL	5	<DL	6.7	10.1
0.05	0.21	12	84.3	<DL	5	<DL	5.2	5.1
0.08	0.12	4.3	63.9	<DL	5	<DL	6.2	10.8
0	0.36	7.6	80.8	<DL	5	<DL	6.2	6
0.07	0.18	7.9	348	<DL	5	<DL	9	3.6
0	0.08	19.4	21.8	<DL	5	<DL	5.3	5.6
0	0	10.3	6.9	<DL	5	<DL	9	6.1
0	0	8.3	442	<DL	5	<DL	8	6.8
0.08	0.24	4.3	182	<DL	5	<DL	8.1	4.6
0	0	4.1	27.4	<DL	5	<DL	2.5	11.1
0.08	0.29	7.8	486	<DL	5	<DL	5.4	8.3
0	0.04	7.6	28.6	<DL	5	<DL	2.5	14.7
0	0.06	3.5	64.9	<DL	5	<DL	5.8	7.2

IL-6	CD8(+) CD4(+)	CD8(+) CD4(-)	CD8(-) CD4(+)	CD8(-) CD4(-)	CD4/CD8ratio
1.9	1.5	37.7	45.1	15.7	1.19
1.3	1.5	24.2	38.7	35.6	1.56
1.5	0.9	23.9	59.1	16.1	2.42
1.3	0.4	15.2	62.2	22.2	4.01
1.5	0.6	26.1	52.1	21.2	1.97
0.8	1.7	18.1	57.8	22.4	3.01
1.6	5.5	23.9	52.8	17.8	1.98
0.8	1.1	26.8	48.4	23.7	1.77
0.9	0.4	30.6	31.8	37.2	1.04
1.2	0.6	28.4	49.3	21.7	1.72
1.1	1.1	30	53.8	15.1	1.77
2.6	2	24.5	53.6	19.9	2.1
41.2	1.9	35.1	43.7	19.3	1.23
0.8	0.8	26.4	47.3	25.5	1.77
0.6	1	33.2	47.4	18.4	1.42
1.4	1.3	25.5	46.8	26.4	1.79
1.2	0.6	25.3	47.7	26.4	1.86
0.8	1.4	23.9	47.2	27.5	1.92
18.6	2.2	14.5	49.8	33.5	3.11
0.9	0.8	19.3	54.6	25.3	2.76
